# Guidelines for the diagnosis and management of post-TB lung disease: a call to action

**DOI:** 10.5588/ijtldopen.24.0093

**Published:** 2024-03-01

**Authors:** D.R. Silva, S. Inwentarz

**Affiliations:** ^1^ Faculdade de Medicina, Universidade Federal do Rio Grande do Sul (UFRGS), Porto Alegre, RS, Brazil;; ^2^ Instituto Vaccarezza, Facultad de Medicina, Universidad de Buenos Aires, Buenos Aires, Argentina

**Keywords:** sequelae, advocacy, guidelines, Mycobacterium tuberculosis, lung disease

More than 10 million people worldwide fall ill with TB each year. In 2022, TB was the second leading infectious killer (after COVID-19), and 1.3 million people died from TB.^1^ However, TB is preventable and usually curable. Treatment success rates have increased to 88% for drug-susceptible TB and 63% for multidrug-resistant TB/rifampicin-resistant TB (MDR/RR-TB).^1^ However, even after TB treatment completion, monitoring and clinical/functional assessment remain essential, as approximately one third of patients surviving pulmonary TB have post-TB lung disease (PTLD). PTLD is defined as ‘evidence of chronic respiratory abnormality, with or without symptoms, attributable at least in part to previous (pulmonary) TB.’^2–5^ PTLD includes mild to moderate disorders such as bronchiectasis, fibrosis, pleural thickening, cavitation, pulmonary hypertension and colonisation and infection with *Aspergillus fumigatus* and non-tuberculous mycobacteria.^2,4,6–11^ Patients with PTLD are 2x more likely than the general population to have spirometry abnormalities, and approximately 10% have lost more than 50% of lung function.^12–14^ Furthermore, mortality is up to 3-to 6-fold higher in PTLD patients than in the general population,^15–17^ with most deaths occurring in the first year after treatment completion.^18^ In fact, patients surviving TB face considerable ongoing morbidities besides PTLD, such as cardiac disorders, neurological impairments, psychosocial challenges and reduced health-related quality of life.^19^

In this context, this issue of the *Journal* includes a paper by Allwood et al., which presents a summary of the proceedings of the 2^nd^ International Post-TB Symposium, held in Stellenbosch, South Africa.^20^ The article describes what was discussed and the goals of 10 academic working groups (including those focused on PTLD, cardiovascular, neuromuscular/skeletal and paediatric complications, among others). The groups identified research priorities, updated knowledge about post-TB sequelae and developed research collaborations. Such a document is important as there are an estimated 155 million TB survivors alive globally, a significant proportion of whom have different types of sequelae. Given the estimated burden of TB sequelae, we urgently need to transform words into action. National societies and national TB programmes need to develop guidelines addressing the diagnosis and management of TB sequelae, especially PTLD. In this context, the Brazilian Respiratory Society (*Sociedade Brasileira de Pneumologia e Tisiologia*, or SBPT) have developed recommendations for the management of PTLD,^21^ adapted from the latest Clinical Standards^22^ for Brazil. A group of 11 pulmonologists and one methodologist participated in the process. Three panel members initially reviewed the current evidence on PTLD and drafted the recommendations, with sections on definition and prevalence of PTLD, assessment of PTLD and management of PTLD. All panel members then reviewed the recommendations until a consensus was reached. The document was formally approved during the 2023 SBPT Annual Conference and published in December 2023. Similarly, the Latin American Thoracic Association (*Asociación Latinoamericana de Tórax* or ALAT), has created ‘Recommendations for the Management of Post-Tuberculosis Lung Disease, PTLD’ (which will soon be published), whose objective is to promote early detection in adults and children, and improve the care of patients with PTLD and their quality of life. Furthermore, the ALAT guidelines will guide physicians, national programme and public health coordinators in planning and implementing appropriate measures to prevent and manage PTLD. Special attention is given to the vaccination status of this patient population. The ALAT guidelines promote the integration and continuum of care of patients within the national TB programmes to achieve the best possible short- and long-term health status for PTLD patients in Latin America – and prevent catastrophic costs.

In conclusion, ensuring quality diagnosis, treatment and prevention of PTLD according to locally tailored recommendations – and with adequate funding – is key to supporting healthcare professionals and policymakers in tackling this clinical and public health priority.
